# Fourier series of sums of products of ordered Bell and poly-Bernoulli functions

**DOI:** 10.1186/s13660-017-1359-2

**Published:** 2017-04-28

**Authors:** Taekyun Kim, Dae San Kim, Dmitry V Dolgy, Jin-Woo Park

**Affiliations:** 1 Department of Mathematics, College of Science, Tianjin Polytechnic University, Tianjin, 300160 China; 2 Department of Mathematics, Kwangwoon University, Seoul, 139-701 Republic of Korea; 3 Department of Mathematics, Sogang University, Seoul, 121-742 Republic of Korea; 4 Hanrimwon, Kwangwoon University, Seoul, 139-701 Republic of Korea; 5 Department of Mathematics Education, Daegu University, Gyeongsan-si, Gyeongsangbuk-do 712-714 Republic of Korea

**Keywords:** 11B68, 11B83, 42A16, Fourier series, ordered Bell function, poly-Bernoulli function

## Abstract

In this paper, we study three types of sums of products of ordered Bell and poly-Bernoulli functions and derive their Fourier series expansion. In addition, we express those functions in terms of Bernoulli functions.

## Introduction

The *ordered Bell polynomials* $b_{m}(x)$ are defined by the generating function

1 $$ \frac{1}{2-e^{t}}e^{xt}=\sum_{m=0} ^{\infty}b_{m}(x)\frac{t^{m}}{m!}. $$

Thus they form an Appell sequence. For $x=0$, $b_{m}=b_{m}(0)$, $(m\geq0)$ are called *ordered Bell numbers* which have been studied in various counting problems in number theory and enumerative combinatorics (see [1, 4, 5, 16, 17, 19]). The ordered Bell numbers are all positive integers, as we can see, for example, from

$$ b_{m}=\sum_{n=0} ^{m}n!S_{2}(m,n)= \sum_{n=0} ^{\infty}\frac {n^{m}}{2^{n+1}}\quad(m \geq0). $$

The first few ordered Bell polynomials are as follows:

$$\begin{aligned}& b_{0}(x)=1,\qquad b_{1}(x)=x+1,\qquad b_{2}(x)=x^{2}+2x+3, \\& b_{3}(x)=x^{3}+3x^{2}+9x+13,\qquad b_{4}(x)=x^{4}+4x^{3}+18x^{2}+52x+75, \\& b_{5}(x)=x^{5}+5x^{4}+30x^{3}+130x^{2}+375x+541. \end{aligned}$$

From (1), we can derive

$$\begin{aligned}& \frac{d}{dx}b_{m}(x)=mb_{m-1}(x)\quad(m\geq1), \\& -b_{m}(x+1)+2b_{m}(x)=x^{m}\quad(m\geq0). \end{aligned}$$

From these, in turn, we have

$$\begin{aligned}& -b_{m}(1)+2b_{m}=\delta_{m,0}\quad(m\geq0), \\& \int_{0} ^{1} b_{m}(x)\,dx= \frac{1}{m+1} \bigl(b_{m+1}(1)-m_{m+1} \bigr) \\& \phantom{int_{0} ^{1} b_{m}(x)\,dx}=\frac{1}{m+1}b_{m+1}. \end{aligned}$$

For any integer *r*, the *poly-Bernoulli polynomials of index* *r* ${\mathbb{B}}_{m} ^{(r)}(x)$ are given by the generating function (see [2, 3, 7–10, 12, 13, 20])

$$ \frac{Li_{r} (1-e^{-t} )}{e^{t}-1}e^{xt}=\sum_{m=0} ^{\infty }{\mathbb{B}}_{m} ^{(r)}(x) \frac{t^{m}}{m!}, $$

where $Li_{r}(x)=\sum_{m=1} ^{\infty}\frac{x^{m}}{m^{r}}$ is the polylogarithmic function for $r\geq1$ and a rational function for $r\leq0$.

We note here that

$$ \frac{d}{dx} \bigl(Li_{r+1}(x) \bigr)=\frac{1}{x}Li_{r}(x). $$

Also, we need the following for later use.

$$\begin{aligned}& \frac{d}{dx}\mathbb{B}_{m}^{(r)}(x)=m{ \mathbb{B}}_{m-1}^{(r)}(x)\quad (m\geq1), \\& \mathbb{B}_{m}^{(1)}(x)=B_{m}(x),\qquad{ \mathbb{B}}_{0} ^{(r)}(x)=1,\qquad \mathbb {B}_{m}^{(0)}(x)=x^{m}, \\& \mathbb{B}_{m}^{(0)}=\delta_{m,0},\qquad\mathbb {B}_{m}^{(r+1)}(1)-\mathbb {B}_{m}^{(r+1)}(0)={ \mathbb{B}}_{m-1} ^{(r)}\quad(m\geq1), \\& \int_{0} ^{1}\mathbb{B}_{m}^{(r)}(x) \,dx=\frac{1}{m+1} \bigl({\mathbb {B}}_{m+1} ^{(r)}(1)-{ \mathbb{B}}_{m+1} ^{(r)}(0) \bigr) \\& \phantom{\int_{0} ^{1}\mathbb{B}_{m}^{(r)}(x) \,dx}=\frac{1}{m+1}\mathbb{B}_{m}^{(r-1)}. \end{aligned}$$

Here the *Bernoulli polynomials* $B_{m}(x)$ are given by the generating function

$$ \frac{t}{e^{t}-1}e^{xt}=\sum_{m=0} ^{\infty}B_{m}(x)\frac{t^{m}}{m!}. $$

For any real number *x*, we let

$$ \langle x \rangle=x-\lfloor x \rfloor\in[0,1) $$

denote the fractional part of *x*.

Finally, we recall the following facts about Bernoulli functions $B_{m}( \langle x \rangle)$:

- for $m\geq2$,
  $$ B_{m}\bigl( \langle x \rangle\bigr)=-m!\sum _{\substack{n=-\infty\\ n\neq0}} ^{\infty}\frac{e^{2\pi inx}}{(2\pi in)^{m}}; $$
- for $m=1$,
  $$ -\sum_{\substack{n=-\infty\\n\neq0}} ^{\infty}\frac{e^{2\pi inx}}{2\pi in}= \textstyle\begin{cases} B_{1}( \langle x \rangle),& \text{for }x\notin{\mathbb {Z}},\\ 0,& \text{for } x\in \mathbb{Z}. \end{cases} $$

Here we will study three types of sums of products of ordered Bell and poly-Bernoulli functions and derive their Fourier series expansion. In addition, we will express those functions in terms of Bernoulli functions.

1. $\alpha_{m}( \langle x \rangle)=\sum_{k=0} ^{m}b_{k}( \langle x \rangle){\mathbb{B}}_{m-k} ^{(r+1)}( \langle x \rangle), (m\geq1)$;
2. $\beta_{m}( \langle x \rangle)=\sum_{k=0} ^{m} \frac {1}{k!(m-k)!}b_{k}( \langle x \rangle){\mathbb{B}}_{m-k} ^{(r+1)}( \langle x \rangle), (m \geq1)$;
3. $\gamma_{m}( \langle x \rangle)=\sum_{k=1} ^{m-1}\frac {1}{k(m-k)}b_{k}( \langle x \rangle){\mathbb{B}}_{m-k} ^{(r+1)}( \langle x \rangle), (m \geq2)$.

For elementary facts about Fourier analysis, the reader may refer to any book (for example, see [18, 21]).

As to $\gamma_{m}(\langle x \rangle)$, we note that the next polynomial identity follows immediately from Theorems 4.1 and 4.2, which is in turn derived from the Fourier series expansion of $\gamma_{m}( \langle x \rangle)$:

$$\begin{aligned}& \sum_{k=1} ^{m-1}\frac{1}{k(m-k)}b_{k}(x){ \mathbb{B}}_{m-k} ^{(r+1)}(x) \\& \quad = \frac{1}{m}\sum_{s=0} ^{m} \binom{m}{s} \biggl(\Lambda _{m-s+1}+\frac {H_{m-1}-H_{m-s}}{m-s+1} \bigl({ \mathbb{B}}_{m-s} ^{(r)}+b_{m-s+1} \bigr) \biggr)B_{s}(x), \end{aligned}$$

where $H_{l}=\sum_{j=1}^{l}\frac{1}{j}$ are the harmonic numbers and

$$ \Lambda_{l}=\sum_{k=1} ^{l-1} \frac{1}{k(l-k)}b_{k}\mathbb {B}_{l-k}^{(r+1)}+2\sum _{k=1} ^{l-1}\frac{1}{k(l-k)}b_{k}{ \mathbb {B}}_{l-k-1} ^{(r)}, $$

with $\Lambda_{1}=0$.

The polynomial identities can be derived also for the functions $\alpha _{m}(\langle x \rangle)$ and $\beta_{m} ( \langle x \rangle)$ from Theorems 2.1 and 2.2, and Theorems 3.1 and 3.2, respectively. We refer the reader to [6, 11, 14, 15] for the recent papers on related works.

## Fourier series of functions of the first type

Let

$$ \alpha_{m}(x)=\sum_{k=0} ^{m} b_{k}(x){\mathbb{B}}_{m-k} ^{(r+1)}(x), $$

where $r,m$ are integers with $m\geq1$. Then we will study the function

$$ \alpha_{m}\bigl( \langle x \rangle\bigr)=\sum _{k=0} ^{m}b_{k}\bigl( \langle x \rangle \bigr){\mathbb{B}}_{m-k} ^{(r+1)}\bigl( \langle x \rangle\bigr) \quad(m\geq1), $$

defined on ${\mathbb{R}}$ which is periodic with period 1.

The Fourier series of $\alpha_{m}( \langle x \rangle)$ is

$$ \sum_{n=-\infty} ^{\infty} A_{n} ^{(m)}e^{2\pi inx}, $$

where

$$\begin{aligned} A_{n} ^{(m)} =& \int_{0} ^{1} \alpha_{m}\bigl( \langle x \rangle \bigr)e^{-2\pi inx}\,dx \\ =& \int_{0} ^{1} \alpha_{m} (x)e^{-2\pi inx}\,dx. \end{aligned}$$

Before proceeding further, we observe the following

$$\begin{aligned} \alpha_{m}'(x) =&\sum _{k=0} ^{m} \bigl\{ kb_{k-1}(x){ \mathbb{B}}_{m-k} ^{(r+1)}(x)+(m-k)b_{k}{\mathbb {B}}_{m-k-1} ^{(r+1)}(x) \bigr\} \\ =&\sum_{k=1} ^{m} kb_{k-1}(x){ \mathbb{B}}_{m-k} ^{(r+1)}(x)+\sum_{k=0} ^{m-1}(m-k)b_{k}(x){\mathbb{B}}_{m-k-1} ^{(r+1)}(x) \\ =&\sum_{k=0} ^{m-1}(k+1)b_{k}(x){ \mathbb{B}}_{m-k-1} ^{(r+1)}(x)+\sum_{k=0} ^{m-1}(m-k)b_{k}(x){\mathbb{B}}_{m-k-1} ^{(r+1)}(x) \\ =&(m+1)\alpha_{m-1}(x). \end{aligned}$$

Thus $(\frac{\alpha_{m+1}(x)}{m+2} )'=\alpha_{m}(x)$, and so $\int_{0} ^{1}\alpha_{m} (x)\,dx=\frac{1}{m+2} (\alpha_{m+1}(1)-\alpha _{m+1}(0) )$. For $m\geq1$, we put

$$\begin{aligned} \Delta_{m} =&\alpha_{m}(1)- \alpha_{m}(0) \\ =&\sum_{k=0} ^{m} b_{k}(1){ \mathbb{B}}_{m-k} ^{(r+1)}(1)-\sum_{k=0} ^{m} b_{k}{\mathbb{B}}_{m-k} ^{(r+1)} \\ =&\sum_{k=0} ^{m-1} (2b_{k}- \delta_{k,0} ) \bigl({\mathbb {B}}_{m-k} ^{(r+1)}+{ \mathbb {B}}_{m-k-1} ^{(r)} \bigr)+2b_{m}- \delta_{m,0}-\sum_{k=0} ^{m}b_{k}{ \mathbb{B}}_{m-k} ^{(r+1)} \\ =&2\sum_{k=0} ^{m-1}b_{k}{ \mathbb{B}}_{m-k} ^{(r+1)}+2\sum_{k=0} ^{m-1}b_{k}{\mathbb{B}}_{m-k-1} ^{(r)}-{ \mathbb{B}}_{m}^{(r+1)}-{\mathbb{B}}_{m-1} ^{(r)}+b_{m}-\sum_{k=0} ^{m-1}b_{k}{\mathbb{B}}_{m-k} ^{(r+1)} \\ =&\sum_{k=1} ^{m} b_{k}{ \mathbb{B}}_{m-k} ^{(r+1)}+2\sum_{k=0} ^{m-1}b_{k}{\mathbb{B}}_{m-k-1} ^{(r)}-{ \mathbb{B}}_{m-1} ^{(r)}. \end{aligned}$$

Thus, $\alpha_{m}(0)=\alpha_{m}(1) \Longleftrightarrow\Delta_{m}=0$ and $\int _{0} ^{1} \alpha_{m}(x)\,dx=\frac{1}{m+2}\Delta_{m+1}$.

Now, we want to determine the Fourier coefficients $A_{n} ^{(m)}$.

*Case* 1: $n\neq0$.

$$\begin{aligned} A_{n} ^{(m)} =& \int_{0} ^{1} \alpha_{m}(x)e^{-2\pi inx} \,dx \\ =&-\frac{1}{2\pi in} \bigl[\alpha_{m}(x)e^{-2\pi inx} \bigr]_{0} ^{1}+\frac {1}{2\pi in} \int_{0} ^{1} \alpha_{m}'(x)e^{-2\pi inx} \,dx \\ =&\frac{m+1}{2\pi in} \int_{0} ^{1} \alpha_{m-1}(x)e^{-2\pi inx} \,dx-\frac {1}{2\pi in} \bigl(\alpha_{m}(1)-\alpha_{m}(0) \bigr) \\ =&\frac{m+1}{2\pi in}A_{n} ^{(m-1)}-\frac{1}{2\pi in} \Delta_{m} \\ =&\frac{m+1}{2\pi in} \biggl(\frac{m}{2\pi in}A_{n} ^{(m-2)}- \frac {1}{2\pi in}\Delta_{m-1} \biggr)-\frac{1}{2\pi in} \Delta_{m} \\ =&\frac{(m+1)_{2}}{(2\pi in)^{2}}A_{n} ^{(m-2)}-\sum _{j=1} ^{2}\frac {(m+1)_{j-1}}{(2\pi in)^{j}}\Delta_{m-j+1} \\ =&\cdots \\ =&\frac{(m+1)_{m}}{(2\pi in)^{m}}A_{n} ^{(0)}-\sum _{j=1} ^{m} \frac {(m+1)_{j-1}}{(2\pi in)^{j}}\Delta_{m-j+1} \\ =&-\frac{1}{m+2}\sum_{j=1} ^{m} \frac{(m+2)_{j}}{(2\pi in)^{j}}\Delta_{m-j+1}, \end{aligned}$$

where we note that $A_{n}^{(0)}=\int_{0}^{1}e^{-2 \pi inx}\,dx=0$.

*Case* 2: $n=0$.

$$ A_{0} ^{(m)}= \int_{0} ^{1}\alpha_{m}(x)\,dx= \frac{1}{m+2}\Delta_{m+1}. $$

$\alpha_{m}( \langle x \rangle)$, $(m\geq1)$ is piecewise $C^{\infty}$. Moreover, $\alpha _{m}( \langle x \rangle)$ is continuous for those positive integers *m* with $\Delta_{m}=0$ and discontinuous with jump discontinuities at integers for those positive integers *m* with $\Delta_{m}\neq0$.

Assume first that *m* is a positive integer with $\Delta_{m}=0$. Then $\alpha_{m}(0)=\alpha_{m}(1)$. Hence $\alpha_{m}( \langle x \rangle)$ is piecewise $C^{\infty }$ and continuous. Thus the Fourier series of $\alpha_{m}( \langle x \rangle)$ converges uniformly to $\alpha_{m}( \langle x \rangle)$, and

$$\begin{aligned} \alpha_{m}\bigl( \langle x \rangle\bigr) =& \frac{1}{m+2}\Delta _{m+1}+\sum_{\substack{n=-\infty\\n\neq 0}} ^{\infty} \Biggl(-\frac{1}{m+2}\sum_{j=1} ^{m}\frac{(m+2)_{j}}{(2\pi in)^{j}}\Delta_{m-j+1} \Biggr)e^{2\pi inx} \\ =&\frac{1}{m+2}\Delta_{m+1}+\frac{1}{m+2}\sum _{j=1} ^{m}\binom {m+2}{j}\Delta_{m-j+1} \Biggl(-j!\sum_{\substack{n=-\infty\\n\neq0}} ^{\infty}\frac{e^{2\pi inx}}{(2\pi in)^{j}} \Biggr) \\ =&\frac{1}{m+2}\Delta_{m+1}+\frac{1}{m+2}\sum _{j=2} ^{m} \binom {m+2}{j}\Delta_{m-j+1}B_{j} \bigl( \langle x \rangle\bigr) \\ &{}+\Delta_{m}\times \textstyle\begin{cases} B_{1}( \langle x \rangle),& \text{for } x\notin \mathbb{Z},\\ 0,& \text{for } x\in \mathbb{Z}. \end{cases}\displaystyle \end{aligned}$$

Now, we can state our first theorem.

### Theorem 2.1

*For each positive integer* *l*, *we let*

$$ \Delta_{l}=\sum_{k=1} ^{l} b_{k}{\mathbb{B}}_{l-k} ^{(r+1)}+2\sum _{k=0} ^{l-1}b_{k}{\mathbb{B}}_{l-k-1} ^{(r)}-{\mathbb{B}}_{l-1} ^{(r)}. $$

*Assume that* $\Delta_{m}=0$ *for a positive integer* *m*. *Then we have the following*

- $\sum_{k=0} ^{m} b_{k}( \langle x \rangle ){\mathbb{B}}_{m-k} ^{(r+1)}( \langle x \rangle)$ *has the Fourier series expansion*
  $$\begin{aligned}& \sum_{k=0} ^{m} b_{k} \bigl( \langle x \rangle\bigr){\mathbb{B}}_{m-k} ^{(r+1)}\bigl( \langle x \rangle\bigr) \\& \quad=\frac{1}{m+2}\Delta_{m+1}+\sum_{\substack{n=-\infty\\n\neq 0}}^{\infty } \Biggl(-\frac{1}{m+2}\sum_{j=1} ^{m} \frac{(m+2)_{j}}{(2\pi in)^{j}}\Delta _{m-j+1} \Biggr)e^{2\pi inx}, \end{aligned}$$
  *for all* $x\in{\mathbb{R}}$, *where the convergence is uniform*.
- $$ \sum_{k=0} ^{m} b_{k}\bigl( \langle x \rangle\bigr){\mathbb{B}}_{m-k} ^{(r+1)}\bigl( \langle x \rangle\bigr)=\frac{1}{m+2}\Delta _{m+1}+\frac {1}{m+2}\sum _{j=2} ^{m} \binom{m+2}{j} \Delta_{m-j+1}B_{j}\bigl( \langle x \rangle\bigr), $$
  *for all* $x\in{\mathbb{R}}$, *where* $B_{j}( \langle x \rangle )$ *is the Bernoulli function*.

Assume next that $\Delta_{m}\neq0$ for a positive integer *m*. Then $\alpha_{m}(0)\neq\alpha_{m}(1)$. So $\alpha_{m}( \langle x \rangle)$ is piecewise $C^{\infty }$ and discontinuous with jump discontinuities at integers. The Fourier series of $\alpha_{m}( \langle x \rangle)$ converges pointwise to $\alpha_{m}( \langle x \rangle)$, for $x\notin{\mathbb{Z}}$, and converges to

$$ \frac{1}{2} \bigl(\alpha_{m}(0)+\alpha_{m}(1) \bigr)= \alpha_{m}(0)+\frac {1}{2}\Delta_{m}, $$

for $x\in{\mathbb{Z}}$.

Now, we can state our second theorem.

### Theorem 2.2

*For each positive integer* *l*, *we let*

$$ \Delta_{l}=\sum_{k=1} ^{l} b_{k}\mathbb{B}_{l-k}^{(r+1)}+2\sum _{k=0} ^{l-1}b_{k}{\mathbb{B}}_{l-k-1} ^{(r)}-{\mathbb{B}}_{l-1} ^{(r)}. $$

*Assume that* $\Delta_{m}\neq0$ *for a positive integer* *m*. *Then we have the following*.

- $$\begin{aligned}& \frac{1}{m+2}\Delta_{m+1}+\sum _{\substack{n=-\infty\\n\neq 0}}^{\infty } \Biggl(-\frac{1}{m+2}\sum _{j=1} ^{m}\frac{(m+2)_{j}}{(2\pi in)^{j}}\Delta _{m-j+1} \Biggr)e^{2\pi inx} \\& \quad= \textstyle\begin{cases} \sum_{k=0} ^{m} b_{k}( \langle x \rangle){\mathbb{B}}_{m-k} ^{(r+1)}( \langle x \rangle),& \textit{for } x\notin {\mathbb{Z}},\\ \sum_{k=0} ^{m} b_{k}{\mathbb{B}}_{m-k} ^{(r+1)}+\frac{1}{2}\Delta _{m},& \textit{for } x\in{\mathbb{Z}}; \end{cases}\displaystyle \end{aligned}$$
- $$\begin{aligned}& \frac{1}{m+2}\Delta_{m+1}+\frac{1}{m+2}\sum _{j=1} ^{m}\binom {m+2}{j} \Delta_{m-j+1}B_{j}\bigl( \langle x \rangle\bigr) \\& \quad=\sum_{k=0} ^{m}b_{k}\bigl( \langle x \rangle\bigr){\mathbb{B}}_{m-k} ^{(r+1)}\bigl( \langle x \rangle\bigr),\quad \textit{for } x\notin {\mathbb{Z}}; \\& \frac{1}{m+2}\Delta_{m+1}+\frac{1}{m+2}\sum _{j=2} ^{m}\binom {m+2}{j}\Delta_{m-j+1}B_{j} \bigl( \langle x \rangle\bigr) \\& \quad=\sum_{k=0} ^{m}b_{k}{ \mathbb{B}}_{m-k} ^{(r+1)}+\frac{1}{2}\Delta _{m},\quad \textit{for } x\in{\mathbb{Z}}. \end{aligned}$$

## Fourier series of functions of the second type

Let $\beta_{m}(x)=\sum_{k=0} ^{m} \frac{1}{k!(m-k)!}b_{k}(x){\mathbb {B}}_{m-k} ^{(r+1)}(x)$, where $r,m$ are integers with $m\geq1$. Then we will investigate the function

$$ \beta_{m}\bigl( \langle x \rangle\bigr)=\sum _{k=0} ^{m} \frac {1}{k!(m-k)!}b_{k}\bigl( \langle x \rangle\bigr){\mathbb{B}}_{m-k} ^{(r+1)}\bigl( \langle x \rangle\bigr), $$

defined on ${\mathbb{R}}$, which is periodic with period 1.

The Fourier series of $\beta_{m}( \langle x \rangle)$ is

$$ \sum_{n=-\infty} ^{\infty}B_{n} ^{(m)}e^{2\pi inx}, $$

where

$$\begin{aligned} B_{n} ^{(m)} =& \int_{0} ^{1} \beta_{m}\bigl( \langle x \rangle \bigr)e^{-2\pi inx}\,dx \\ =& \int_{0} ^{1} \beta_{m}(x)e^{-2\pi inx} \,dx. \end{aligned}$$

Before proceeding further, we note the following.

$$\begin{aligned} \beta_{m}'(x) =&\sum_{k=0} ^{m} \biggl\{ \frac {k}{k!(m-k)!}b_{k-1}(x){ \mathbb{B}}_{m-k} ^{(r+1)} (x)+\frac{m-k}{k!(m-k)!}b_{k}(x){ \mathbb{B}}_{m-k-1} ^{(r+1)}(x) \biggr\} \\ =&\sum_{k=1} ^{m} \frac{1}{(k-1)!(m-k)!}b_{k-1}(x){ \mathbb{B}}_{m-k} ^{(r+1)}(x)+\sum_{k=0} ^{m-1}\frac{1}{k!(m-k-1)!}b_{k}(x){\mathbb{B}}_{m-k-1} ^{(r+1)}(x) \\ =&\sum_{k=0} ^{m-1}\frac{1}{k!(m-k-1)!}b_{k}(x){ \mathbb{B}}_{m-k-1} ^{(r+1)}(x)+\sum_{k=0} ^{m-1}\frac{1}{k!(m-1-k)!}b_{k}(x){\mathbb {B}}_{m-k-1} ^{(r+1)}(x) \\ =&2\beta_{m-1}(x). \end{aligned}$$

Thus

$$ \biggl(\frac{\beta_{m+1}(x)}{2} \biggr)'=\beta_{m}(x),\quad \text{and}\quad \int_{0} ^{1} \beta_{m}(x)\,dx= \frac{1}{2}\bigl(\beta_{m+1}(1)-\beta_{m+1}(0)\bigr). $$

For $m\geq1$, we put

$$\begin{aligned} \Omega_{m} =&\beta_{m}(1)-\beta_{m}(0) \\ =&\sum_{k=0} ^{m}\frac{1}{k!(m-k)!}b_{k}(1){ \mathbb{B}}_{m-k} ^{(r+1)}(1)-\sum_{k=0} ^{m}\frac {1}{k!(m-k)!}b_{k}{\mathbb{B}}_{m-k} ^{(r+1)} \\ =&\sum_{k=0} ^{m-1}\frac{1}{k!(m-k)!}(2b_{k}- \delta_{k,0}) \bigl({\mathbb{B}}_{m-k} ^{(r+1)} +{ \mathbb{B}}_{m-k-1} ^{(r)} \bigr) \\ &{}+\frac{1}{m!}(2b_{m}-\delta_{m,0})-\sum _{k=0} ^{m}\frac {1}{k!(m-k)!}b_{k}{ \mathbb{B}}_{m-k} ^{(r+1)} \\ =& 2\sum_{k=0} ^{m-1}\frac{1}{k!(m-k)!}b_{k}{ \mathbb{B}}_{m-k} ^{(r+1)}+2\sum_{k=0} ^{m-1}\frac {1}{k!(m-k)!}b_{k}{\mathbb{B}}_{m-k-1} ^{(r)} \\ &{}-\frac{1}{m!}{\mathbb{B}}_{m}^{(r+1)}- \frac{1}{m!}{\mathbb{B}}_{m-1} ^{(r)} + \frac {1}{m!}b_{m}-\sum_{k=0} ^{m-1}\frac{1}{k!(m-k)!}b_{k}{\mathbb{B}}_{m-k} ^{(r+1)} \\ =&\sum_{k=1} ^{m} \frac{1}{k!(m-k)!}b_{k}{ \mathbb{B}}_{m-k} ^{(r+1)}+2\sum_{k=0} ^{m-1}\frac {1}{k!(m-k)!}b_{k}{\mathbb{B}}_{m-k-1} ^{(r)}-\frac{1}{m!}{\mathbb {B}}_{m-1} ^{(r)}. \end{aligned}$$

Hence

$$ \beta_{m}(0)=\beta_{m}(1)\quad\Longleftrightarrow\quad \Omega_{m}=0,\quad\text{and}\quad \int _{0} ^{1} \beta_{m}(x)\,dx= \frac{1}{2}\Omega_{m+1}. $$

We now would like to determine the Fourier coefficients $B_{n} ^{(m)}$.

*Case* 1: $n\neq0$.

$$\begin{aligned} B_{n} ^{(m)} =& \int_{0} ^{1} \beta_{m} (x)e^{-2\pi inx}\,dx \\ =&-\frac{1}{2\pi in} \bigl[\beta_{m}(x)e^{-2\pi inx} \bigr]_{0} ^{1}+\frac {1}{2\pi in} \int_{0} ^{1} \beta_{m} '(x)e^{-2\pi inx}\,dx \\ =&-\frac{1}{2\pi in} \bigl(\beta_{m}(1)-\beta_{m}(0) \bigr)+\frac {2}{2\pi in} \int_{0} ^{1} \beta_{m-1}(x)e^{-2\pi inx} \,dx \\ =&\frac{2}{2\pi in}B_{n} ^{(m-1)}-\frac{1}{2\pi in} \Omega_{m} \\ =&\frac{2}{2\pi in} \biggl(\frac{2}{2\pi in}B_{n} ^{(m-2)}- \frac {1}{2\pi in}\Omega_{m-1} \biggr)-\frac{1}{2\pi in} \Omega_{m} \\ =& \biggl(\frac{2}{2\pi in} \biggr)^{2}B_{n} ^{(m-2)}-\sum_{j=1} ^{2} \frac {2^{j-1}}{(2\pi in)^{j}}\Omega_{m-j+1} \\ =&\cdots \\ =& \biggl(\frac{2}{2\pi in} \biggr)^{m}B_{n} ^{(0)}-\sum_{j=1} ^{m} \frac {2^{j-1}}{(2\pi in)^{j}}\Omega_{m-j+1} \\ =&-\sum_{j=1} ^{m} \frac{2^{j-1}}{(2\pi in)^{j}} \Omega_{m-j+1}. \end{aligned}$$

*Case* 2: $n=0$.

$$ B_{0} ^{(m)}= \int_{0} ^{1} \beta_{m}(x)\,dx= \frac{1}{2}\Omega_{m+1}. $$

$\beta_{m}( \langle x \rangle)$, $(m\geq1)$ is piecewise $C^{\infty}$. Moreover, $\beta _{m}( \langle x \rangle)$ is continuous for those positive integers *m* with $\Omega_{m}=0$ and discontinuous with jump discontinuities at integers for those positive integers *m* with $\Omega_{m}\neq0$.

Assume first that *m* is a positive integer with $\Omega_{m}=0$. Then $\beta_{m}(0)=\beta_{m}(1)$. Thus $\beta_{m}( \langle x \rangle)$ is piecewise $C^{\infty}$ and continuous. Hence the Fourier series of $\beta_{m}( \langle x \rangle)$ converges uniformly to $\beta_{m}( \langle x \rangle)$, and

$$\begin{aligned} \beta_{m}\bigl( \langle x \rangle\bigr) =& \frac{1}{2}\Omega _{m+1}+\sum_{\substack{n=-\infty\\n\neq 0}} ^{\infty} \Biggl(-\sum_{j=1} ^{m} \frac{2^{j-1}}{(2\pi in)^{j}}\Omega _{m-j+1} \Biggr)e^{2\pi inx} \\ =&\frac{1}{2}\Omega_{m+1}+\sum_{j=1} ^{m}\frac{2^{j-1}}{j!}\Omega _{m-j+1} \Biggl(-j!\sum _{\substack{n=-\infty\\n\neq0}} ^{\infty} \frac {e^{2\pi inx}}{(2\pi in)^{j}} \Biggr) \\ =&\frac{1}{2}\Omega_{m+1}+\sum_{j=2} ^{m}\frac{2^{j-1}}{j!}\Omega _{m-j+1}B_{j}\bigl( \langle x \rangle\bigr) \\ &{}+\Omega_{m}\times \textstyle\begin{cases} B_{1}( \langle x \rangle),& \text{for } x\notin \mathbb{Z},\\ 0,& \text{for } x\in \mathbb{Z}. \end{cases}\displaystyle \end{aligned}$$

Now, we can state our first result.

### Theorem 3.1

*For each positive integer* *l*, *we let*

$$ \Omega_{l}=\sum_{k=1} ^{l} \frac{1}{k!(l-k)!}b_{k}{\mathbb{B}}_{l-k} ^{(r+1)}+2 \sum_{k=0} ^{l-1}\frac{1}{k!(l-k)!}b_{k}{ \mathbb{B}}_{l-k-1} ^{(r)}-\frac{1}{l!}{ \mathbb{B}}_{l-1} ^{(r)}. $$

*Assume that* $\Omega_{m}=0$ *for a positive integer* *m*. *Then we have the following*.

- $\sum_{k=0} ^{m} \frac{1}{k!(m-k)!}b_{k}( \langle x \rangle){\mathbb{B}}_{m-k} ^{(r+1)}( \langle x \rangle)$ *has the Fourier series expansion*
  $$\begin{aligned}& \sum_{k=0} ^{m} \frac{1}{k!(m-k)!}b_{k}\bigl( \langle x \rangle \bigr){ \mathbb{B}}_{m-k} ^{(r+1)}\bigl( \langle x \rangle\bigr) \\& \quad=\frac{1}{2}\Omega_{m+1}+\sum_{\substack{n=-\infty\\n\neq0}} ^{\infty } \Biggl(-\sum_{j=1} ^{m} \frac{2^{j-1}}{(2\pi in)^{j}}\Omega _{m-j+1} \Biggr)e^{2\pi inx}, \end{aligned}$$
  *for all* $x\in{\mathbb{R}}$, *where the convergence is uniform*.
- $$ \sum_{k=0} ^{m}\frac{1}{k!(m-k)!}b_{k} \bigl( \langle x \rangle \bigr){\mathbb{B}}_{m-k} ^{(r+1)}\bigl( \langle x \rangle\bigr) =\frac{1}{2}\Omega_{m+1}+\sum _{j=2} ^{m} \frac{2^{j-1}}{j!}\Omega _{m-j+1}B_{j}\bigl( \langle x \rangle\bigr), $$
  *for all* $x\in{\mathbb{R}}$, *where* $B_{j}( \langle x \rangle )$ *is the Bernoulli function*.

Assume next that $\Omega_{m}\neq0$ for a positive integer *m*. Then $\beta _{m}(0)\neq\beta_{m}(1)$. So $\beta_{m}( \langle x \rangle)$ is piecewise $C^{\infty}$ and discontinuous with jump discontinuities at integers. The Fourier series of $\beta_{m}( \langle x \rangle)$ converges pointwise to $\beta_{m}( \langle x \rangle)$, for $x\notin {\mathbb{Z}}$, and converges to

$$ \frac{1}{2} \bigl(\beta_{m}(0)+\beta_{m}(1) \bigr)= \beta_{m}(0)+\frac {1}{2}\Omega_{m}, $$

for $x\in{\mathbb{Z}}$.

Now, we can state our second theorem.

### Theorem 3.2

*For each positive integer* *l*, *we let*

$$ \Omega_{l}=\sum_{k=1} ^{l} \frac{1}{k!(l-k)!}b_{k}{\mathbb{B}}_{l-k} ^{(r+1)}+2 \sum_{k=0} ^{l-1}\frac{1}{k!(l-k)!}b_{k}{ \mathbb{B}}_{l-k-1} ^{(r)}-\frac{1}{l!}{ \mathbb{B}}_{l-1} ^{(r)}. $$

*Assume that* $\Omega_{m}\neq0$ *for a positive integer* *m*. *Then we have the following*.

- $$\begin{aligned}& \frac{1}{2}\Omega_{m+1}+\sum_{\substack{n=-\infty\\n\neq0}} ^{\infty } \Biggl(-\sum_{j=1} ^{m} \frac{2^{j-1}}{(2\pi in)^{j}}\Omega _{m-j+1} \Biggr)e^{2\pi inx} \\& \quad= \textstyle\begin{cases} \sum_{k=0} ^{m} \frac{1}{k!(m-k)!}b_{k}( \langle x \rangle ){\mathbb{B}}_{m-k} ^{(r+1)}( \langle x \rangle),& \textit{for } x\notin {\mathbb{Z}},\\ \sum_{k=0} ^{m}\frac{1}{k!(m-k)!}b_{k}{\mathbb{B}}_{m-k} ^{(r+1)}+\frac {1}{2}\Omega_{m},&\textit{for }x\in{\mathbb{Z}}. \end{cases}\displaystyle \end{aligned}$$
- $$\begin{aligned}& \frac{1}{2}\Omega_{m+1}+\sum _{j=1} ^{m}\frac{2^{j-1}}{j!}\Omega _{m-j+1}B_{j}\bigl( \langle x \rangle\bigr) \\& \quad=\sum_{k=0} ^{m}\frac{1}{k!(m-k)!}b_{k} \bigl( \langle x \rangle \bigr){\mathbb{B}}_{m-k} ^{(r+1)}\bigl( \langle x \rangle\bigr),\quad \textit{for } x\notin {\mathbb{Z}}; \\& \frac{1}{2}\Omega_{m+1}+\sum_{j=2} ^{m}\frac{2^{j-1}}{j!}\Omega _{m-j+1}B_{j}\bigl( \langle x \rangle\bigr) \\& \quad=\sum_{k=0} ^{m}\frac{1}{k!(m-k)!}b_{k}{ \mathbb{B}}_{m-k} ^{(r+1)}+\frac{1}{2}\Omega_{m}, \quad \textit{for } x\in{\mathbb{Z}}. \end{aligned}$$

## Fourier series of functions of the third type

Let

$$ \gamma_{m}(x)=\sum_{k=1} ^{m-1} \frac{1}{k(m-k)}b_{k}(x){\mathbb {B}}_{m-k} ^{(r+1)}(x), $$

where $r,m$ are integers with $m\geq2$. Then we will consider the function

$$ \gamma_{m}\bigl( \langle x \rangle\bigr)=\sum _{k=1} ^{m-1}\frac {1}{k(m-k)}b_{k}\bigl( \langle x \rangle\bigr){\mathbb{B}}_{m-k} ^{(r+1)}\bigl( \langle x \rangle\bigr), $$

defined on ${\mathbb{R}}$, which is periodic with period 1.

The Fourier series of $\gamma_{m}( \langle x \rangle)$ is

$$ \sum_{n=-\infty} ^{\infty}C_{n} ^{(m)}e^{2\pi inx}, $$

where

$$\begin{aligned} C_{n} ^{(m)} =& \int_{0} ^{1} \gamma_{m}\bigl( \langle x \rangle \bigr)e^{-2\pi inx}\,dx \\ =& \int_{0} ^{1}\gamma_{m}(x)e^{-2\pi inx} \,dx. \end{aligned}$$

Before proceeding further, we need to observe the following.

$$\begin{aligned} \gamma_{m}'(x) =& \sum _{k=1} ^{m-1}\frac{1}{m-k}b_{k-1}(x){ \mathbb {B}}_{m-k} ^{(r+1)}(x)+\sum_{k=1} ^{m-1}\frac{1}{k}b_{k}(x)\mathbb{B}_{m-k-1}^{(r+1)}(x) \\ =&\sum_{k=0} ^{m-2}\frac{1}{m-k-1}b_{k}(x) \mathbb {B}_{m-k-1}^{(r+1)}(x)+\sum_{k=1} ^{m-1}\frac{1}{k}b_{k}(x){\mathbb {B}}_{m-k-1} ^{(r+1)}(x) \\ =&\sum_{k=1} ^{m-2} \biggl( \frac{1}{m-k-1}+\frac{1}{k} \biggr)b_{k}(x){ \mathbb{B}}_{m-k-1} ^{(r+1)}(x)+\frac{1}{m-1}{ \mathbb{B}}_{m-1} ^{(r+1)}(x)+\frac{1}{m-1}b_{m-1} (x) \\ =&(m-1)\gamma_{m-1}(x)+\frac{1}{m-1}{\mathbb{B}}_{m-1} ^{(r+1)}(x)+\frac {1}{m-1}b_{m-1}(x). \end{aligned}$$

From this, we see that

$$ \biggl(\frac{1}{m} \biggl(\gamma_{m+1}(x)-\frac{1}{m(m+1)}{ \mathbb {B}}_{m+1} ^{(r+1)}(x)-\frac{1}{m(m+1)}b_{m+1} (x) \biggr) \biggr)'=\gamma_{m}(x), $$

and

$$\begin{aligned}& \int_{0} ^{1} \gamma_{m}(x)\,dx \\& \quad=\frac{1}{m} \biggl[\gamma_{m+1}(x)-\frac{1}{m(m+1)}{ \mathbb{B}}_{m+1} ^{(r+1)}(x)-\frac{1}{m(m+1)}b_{m+1}(x) \biggr]_{0} ^{1} \\& \quad=\frac{1}{m}\biggl(\gamma_{m+1}(1)-\gamma_{m}(0)- \frac {1}{m(m+1)} \bigl({\mathbb{B}}_{m+1} ^{(r+1)}(1)-{ \mathbb{B}}_{m+1} ^{(r+1)}(0) \bigr) \\& \qquad{}-\frac{1}{m(m+1)} \bigl(b_{m+1}(1)-b_{m+1}(0) \bigr) \biggr) \\& \quad=\frac{1}{m} \biggl(\gamma_{m+1} (1)-\gamma_{m+1}(0)- \frac {1}{m(m+1)}{\mathbb{B}}_{m} ^{(r)}-\frac{1}{m(m+1)}b_{m+1} \biggr). \end{aligned}$$

For $m\geq2$, we let

$$\begin{aligned} \Lambda_{m} =& \gamma_{m}(1)- \gamma_{m}(0) \\ =&\sum_{k=1} ^{m-1}\frac{1}{k(m-k)} \bigl(b_{k}(1){\mathbb{B}}_{m-k} ^{(r+1)}(1)-b_{k}{ \mathbb{B}}_{m-k} ^{(r+1)} \bigr) \\ =&\sum_{k=1} ^{m-1}\frac{1}{k(m-k)} \bigl((2b_{k}-\delta _{k,0}) \bigl(\mathbb {B}_{m-k}^{(r+1)}+\mathbb{B}_{m-k-1}^{(r)} \bigr)-b_{k}{\mathbb{B}}_{m-k} ^{(r+1)} \bigr) \\ =&\sum_{k=1} ^{m-1}\frac{1}{k(m-k)}b_{k}{ \mathbb{B}}_{m-k} ^{(r+1)}+2\sum_{k=1} ^{m-1}\frac {1}{k(m-k)}b_{k}{\mathbb{B}}_{m-k-1} ^{(r)}. \end{aligned}$$

Then

$$ \gamma_{m}(0)=\gamma_{m}(1)\quad \Longleftrightarrow\quad \Lambda_{m}=0, $$

and

$$ \int_{0} ^{1}\gamma_{m}(x)\,dx= \frac{1}{m} \biggl(\Lambda_{m+1}-\frac {1}{m(m+1)}{ \mathbb{B}}_{m} ^{(r)}-\frac{1}{m(m+1)}b_{m+1} \biggr). $$

Now, we would like to determine the Fourier coefficients $C_{n} ^{(m)}$.

*Case* 1: $n\neq0$. For this computation, we need to know the following:

$$\begin{aligned}& \int_{0} ^{1} {\mathbb{B}}_{l} ^{(r+1)}(x)e^{-2\pi inx}\,dx= \textstyle\begin{cases} -\sum_{k=1} ^{l}\frac{(l)_{k-1}}{(2\pi in)^{k}}{\mathbb{B}}_{l-k} ^{(r)},& \text{for } n\neq0,\\ \frac{1}{l+1}{\mathbb{B}}_{l} ^{(r)},& \text{for } n=0, \end{cases}\displaystyle \\& \int_{0} ^{1}b_{l}(x)e^{-2\pi inx} \,dx= \textstyle\begin{cases} -\sum_{k=1} ^{l} \frac{(l)_{k-1}}{(2\pi in)^{k}}b_{l-k+1},& \text{for }n\neq0,\\ \frac{1}{l+1}b_{l+1},& \text{for } n=0, \end{cases}\displaystyle \\& C_{n} ^{(m)}= \int_{0} ^{1} \gamma_{m}(x)e^{-2\pi inx} \,dx \\& \phantom{C_{n} ^{(m)}}=-\frac{1}{2\pi in} \bigl[\gamma_{m}(x)e^{-2\pi inx} \bigr]_{0} ^{1} +\frac {1}{2\pi in} \int_{0} ^{1} \gamma_{m} '(x)e^{-2\pi inx}\,dx \\& \phantom{C_{n} ^{(m)}}= -\frac{1}{2\pi in} \bigl(\gamma_{m}(1)- \gamma_{m}(0) \bigr) \\& \phantom{C_{n} ^{(m)}=}{}+\frac{1}{2\pi in} \int_{0} ^{1} \biggl((m-1)\gamma_{m-1}(x)+ \frac {1}{m-1}{\mathbb{B}}_{m-1} ^{(r+1)}(x)+ \frac{1}{m-1}b_{m-1}(x) \biggr)e^{-2\pi inx}\,dx \\& \phantom{C_{n} ^{(m)}}=\frac{m-1}{2\pi in}C_{n} ^{(m-1)}- \frac{1}{2\pi in}\Lambda_{m}+\frac {1}{2\pi in(m-1)} \int_{0} ^{1} {\mathbb{B}}_{m-1} ^{(r+1)}(x)e^{-2\pi inx}\,dx \\& \phantom{C_{n} ^{(m)}=}{}+\frac{1}{2\pi in(m-1)} \int_{0} ^{1}b_{m-1}(x)e^{-2\pi inx}\,dx \\& \phantom{C_{n} ^{(m)}}=\frac{m-1}{2\pi in}C_{n} ^{(m-1)}- \frac{1}{2\pi in}\Lambda_{m}-\frac {1}{2\pi in(m-1)}\Theta_{m}- \frac{1}{2\pi in(m-1)}\Phi_{m}, \end{aligned}$$

where

$$\begin{aligned}& \Lambda_{m} = \gamma_{m}(1)-\gamma_{m}(0) \\& \phantom{\Lambda_{m}} = \sum_{k=1} ^{m-1} \frac{1}{k(m-k)}b_{k}{\mathbb{B}}_{m-k} ^{(r+1)}+2 \sum_{k=1} ^{m-1}\frac {1}{k(m-k)}b_{k}{ \mathbb{B}}_{m-k-1} ^{(r)}, \\& \Theta_{m}=\sum_{k=1} ^{m-1} \frac{(m-1)_{k-1}}{(2\pi in)^{k}}{\mathbb {B}}_{m-k-1} ^{(r)},\qquad \Phi_{m}=\sum_{k=1} ^{m-1} \frac{(m-1)_{k-1}}{(2\pi in)^{k}}b_{m-k}. \\& C_{n} ^{(m)}=\frac{m-1}{2\pi in}C_{n} ^{(m-1)}-\frac{1}{2\pi in}\Lambda _{m}-\frac{1}{2\pi in(m-1)} \Theta_{m}-\frac{1}{2\pi in(m-1)}\Phi_{m} \\& \phantom{C_{n} ^{(m)}} = \frac{m-1}{2\pi in} \biggl(\frac{m-2}{2\pi in}C_{n} ^{(m-2)}-\frac {1}{2\pi in}\Lambda_{m-1}-\frac{1}{2\pi in(m-2)} \Theta_{m-1}-\frac {1}{2\pi in(m-2)}\Phi_{m-1} \biggr) \\& \phantom{C_{n} ^{(m)}=}{}-\frac{1}{2\pi in}\Lambda_{m}-\frac{1}{2\pi in(m-1)} \Theta_{m}-\frac {1}{2\pi in(m-1)}\Phi_{m} \\& \phantom{C_{n} ^{(m)}} =\frac{(m-1)_{2}}{(2\pi in)^{2}}C_{n} ^{(m-2)}-\sum _{j=1} ^{2}\frac {(m-1)_{j-1}}{(2\pi in)^{j}}\Lambda_{m-j+1} \\& \phantom{C_{n} ^{(m)}=}{}-\sum_{j=1} ^{2} \frac{(m-1)_{j-1}}{(2\pi in)^{j}(m-j)}\Theta _{m-j+1}-\sum_{j=1} ^{2} \frac{(m-1)_{j-1}}{(2\pi in)^{j}(m-j)}\Phi_{m-j+1} \\& \phantom{C_{n} ^{(m)}}=\cdots \\& \phantom{C_{n} ^{(m)}}=-\sum_{j=1} ^{m-1} \frac{(m-1)_{j-1}}{(2\pi in)^{j}}\Lambda _{m-j+1}-\sum_{j=1} ^{m-1}\frac{(m-1)_{j-1}}{(2\pi in)^{j}(m-j)}\Theta_{m-j+1}-\sum _{j=1} ^{m-1}\frac{(m-1)_{j-1}}{(2\pi in)^{j}(m-j)}\Phi_{m-j+1}. \end{aligned}$$

We note here that

$$\begin{aligned}& \sum_{j=1} ^{m-1} \frac{(m-1)_{j-1}}{(2\pi in)^{j}(m-j)}\Phi_{m-j+1} \\& \quad=\sum_{j=1} ^{m-1}\frac{(m-1)_{j-1}}{(2\pi in)^{j}(m-j)} \sum_{k=1} ^{m-j}\frac{(m-j)_{k-1}}{(2\pi in)^{k}}b_{m-j-k+1} \\& \quad=\sum_{j=1} ^{m-1}\sum _{k=1} ^{m-j}\frac{(m-1)_{j+k-2}}{(2\pi in)^{j+k}(m-j)}b_{m-j-k+1} \\& \quad=\sum_{j=1} ^{m-1}\frac{1}{m-j} \sum_{k=1} ^{m-j} \frac {(m-1)_{j+k-2}}{(2\pi in)^{j+k}}b_{m-j-k+1} \\& \quad=\sum_{j=1} ^{m-1}\frac{1}{m-j} \sum_{s=j+1} ^{m}\frac {(m-1)_{s-2}}{(2\pi in)^{s}}b_{m-s+1} \\& \quad=\sum_{s=2} ^{m} \frac{(m-1)_{s-2}}{(2\pi in)^{s}}b_{m-s+1} \sum_{j=1} ^{s-1}\frac{1}{m-j} \\& \quad= \sum_{s=1} ^{m}\frac{(m-1)_{s-2}}{(2\pi in)^{s}}b_{m-s+1}(H_{m-1}-H_{m-s}) \\& \quad=\frac{1}{m}\sum_{s=1} ^{m} \frac{(m)_{s}}{(2\pi in)^{s}}\frac {H_{m-1}-H_{m-s}}{m-s+1}b_{m-s+1}. \end{aligned}$$

Putting everything together, we get

$$ C_{n} ^{(m)}=-\frac{1}{m}\sum _{s=1} ^{m}\frac{(m)_{s}}{(2\pi in)^{s}} \biggl\{ \Lambda_{m-s+1}+\frac{H_{m-1}-H_{m-s}}{m-s+1} \bigl({\mathbb{B}}_{m-s} ^{(r)}+b_{m-s+1} \bigr) \biggr\} . $$

*Case* 2: $n=0$.

$$\begin{aligned} C_{0} ^{(m)} =& \int_{0} ^{1} \gamma_{m}(x)\,dx \\ =&\frac{1}{m} \biggl(\Lambda_{m+1}-\frac{1}{m(m+1)}{\mathbb {B}}_{m}^{(r)}-\frac {1}{m(m+1)}b_{m+1} \biggr). \end{aligned}$$

$\gamma_{m}( \langle x \rangle)$, $m\geq2$ is piecewise $C^{\infty}$. Moreover, $\gamma _{m}( \langle x \rangle)$ is continuous for those integers $m\geq2$ with $\Lambda_{m}=0$ and discontinuous with jump discontinuities at integers for those integers $m\geq2$ with $\Lambda_{m}\neq0$.

Assume first that $\Lambda_{m}=0$. Then $\gamma_{m}(0)=\gamma_{m}(1)$. Thus $\gamma_{m}( \langle x \rangle)$ is piecewise $C^{\infty}$ and continuous. Hence the Fourier series of $\gamma_{m}( \langle x \rangle)$ converges uniformly to $\gamma_{m}( \langle x \rangle)$, and

$$\begin{aligned} \gamma_{m}\bigl( \langle x \rangle\bigr) =&\frac{1}{m} \biggl(\Lambda_{m+1}-\frac{1}{m(m+1)}{\mathbb {B}}_{m}^{(r)}- \frac {1}{m(m+1)}b_{m+1} \biggr) \\ &{}+\sum_{\substack{n=-\infty\\n\neq0}} ^{\infty} \Biggl\{ - \frac {1}{m}\sum_{s=1} ^{m} \frac{(m)_{s}}{(2\pi in)^{s}} \biggl(\Lambda_{m-s+1}+\frac {H_{m-1}-H_{m-s}}{m-s+1} \bigl({ \mathbb{B}}_{m-s} ^{(r)}+b_{m-s+1} \bigr) \biggr) \Biggr\} e^{2\pi inx} \\ =&\frac{1}{m} \biggl(\Lambda_{m+1}-\frac{1}{m(m+1)}{\mathbb {B}}_{m}^{(r)}-\frac {1}{m(m+1)}b_{m+1} \biggr) \\ &{}+\frac{1}{m}\sum_{s=1} ^{m} \binom{m}{s} \biggl(\Lambda_{m-s+1}+\frac {H_{m-1}-H_{m-s}}{m-s+1} \bigl({ \mathbb{B}}_{m-s} ^{(r)}+b_{m-s+1} \bigr) \biggr) \Biggl(-s!\sum_{\substack{n=-\infty\\n\neq0}} ^{\infty}\frac {e^{2\pi inx}}{(2\pi in)^{s}} \Biggr) \\ =&\frac{1}{m} \biggl(\Lambda_{m+1}-\frac{1}{m(m+1)}{\mathbb {B}}_{m}^{(r)}-\frac {1}{m(m+1)}b_{m+1} \biggr) \\ &{}+\frac{1}{m}\sum_{s=2} ^{m} \binom{m}{s} \biggl(\Lambda_{m-s+1}+\frac {H_{m-1}-H_{m-s}}{m-s+1} \bigl({ \mathbb{B}}_{m-s} ^{(r)}+b_{m-s+1} \bigr) \biggr)B_{s}\bigl( \langle x \rangle\bigr) \\ &{}+\Lambda_{m}\times \textstyle\begin{cases} B_{1}( \langle x \rangle),& \text{for } x\notin{\mathbb {Z}},\\ 0,& \text{for } x\in{\mathbb{Z}} \end{cases}\displaystyle \\ =&\frac{1}{m}\sum_{\substack{s=0\\s\neq1}}^{m} \binom{m}{s} \biggl(\Lambda _{m-s+1}+\frac{H_{m-1}-H_{m-s}}{m-s+1} \bigl({ \mathbb{B}}_{m-s} ^{(r)}+b_{m-s+1} \bigr) \biggr)B_{s}\bigl( \langle x \rangle\bigr) \\ &{}+\Lambda_{m}\times \textstyle\begin{cases} B_{1}( \langle x \rangle),& \text{for } x\notin{\mathbb {Z}},\\ 0,& \text{for } x\in \mathbb{Z}. \end{cases}\displaystyle \end{aligned}$$

Now, we can state our first result.

### Theorem 4.1

*For each integer* $l\geq2$, *we let*

$$ \Lambda_{l}=\sum_{k=1} ^{l-1} \frac{1}{k(l-k)}b_{k}\mathbb {B}_{l-k}^{(r+1)}+2\sum _{k=1} ^{l-1}\frac{1}{k(l-k)}b_{k}{ \mathbb {B}}_{l-k-1} ^{(r)}, $$

*with* $\Lambda_{1}=0$. *Assume that* $\Lambda_{m}=0$ *for an integer* $m\geq2$. *Then we have the following*.

- $\sum_{k=1} ^{m-1}\frac{1}{k(m-k)}b_{k}( \langle x \rangle){\mathbb{B}}_{m-k} ^{(r+1)}( \langle x \rangle)$ *has Fourier series expansion*
  $$\begin{aligned}& \sum_{k=1} ^{m-1} \frac{1}{k(m-k)}b_{k}\bigl( \langle x \rangle \bigr){ \mathbb{B}}_{m-k} ^{(r+1)}\bigl( \langle x \rangle\bigr) \\& \quad=\frac{1}{m} \biggl(\Lambda_{m+1}-\frac{1}{m(m+1)}{ \mathbb {B}}_{m}^{(r)}-\frac {1}{m(m+1)}b_{m+1} \biggr) \\& \qquad{}+\sum_{\substack{n=-\infty\\n\neq0}} ^{\infty} \Biggl\{ - \frac {1}{m}\sum_{s=1} ^{m} \frac{(m)_{s}}{(2\pi in)^{s}} \biggl(\Lambda_{m-s+1}+\frac {H_{m-1}-H_{m-s}}{m-s+1} \bigl({ \mathbb{B}}_{m-s} ^{(r)}+b_{m-s+1} \bigr) \biggr) \Biggr\} e^{2\pi inx}, \end{aligned}$$
  *for all* $x\in{\mathbb{R}}$, *where the convergence is uniform*.
- $$\begin{aligned}& \sum_{k=1} ^{m-1}\frac{1}{k(m-k)}b_{k} \bigl( \langle x \rangle \bigr){\mathbb{B}}_{m-k} ^{(r+1)}\bigl( \langle x \rangle\bigr) \\& \quad=\frac{1}{m}\sum_{\substack{s=0\\s\neq1}} ^{m} \binom{m}{s} \biggl(\Lambda _{m-s+1}+\frac{H_{m-1}-H_{m-s}}{m-s+1} \bigl({ \mathbb{B}}_{m-s} ^{(r)}+b_{m-s+1} \bigr) \biggr)B_{s}\bigl( \langle x \rangle\bigr), \end{aligned}$$
  *for all* $x\in{\mathbb{R}}$, *where* $B_{s}( \langle x \rangle )$ *is the Bernoulli function*.

Assume next that *m* is an integer ≥2 with $\Lambda_{m}\neq0$. Then $\gamma_{m}(0)\neq\gamma_{m}(1)$. Hence $\gamma_{m}( \langle x \rangle)$ is piecewise $C^{\infty}$ and discontinuous with jump discontinuities at integers. Then the Fourier series of $\gamma_{m}( \langle x \rangle)$ converges pointwise to $\gamma _{m}( \langle x \rangle)$, for $x\notin{\mathbb{Z}}$, and converges to

$$\begin{aligned} \frac{1}{2} \bigl(\gamma_{m}(0)+ \gamma_{m}(1) \bigr) =&\gamma _{m}(0)+\frac {1}{2} \Lambda_{m} \\ =&\sum_{k=1} ^{m-1}\frac{1}{k(m-k)}b_{k}{ \mathbb{B}}_{m-k} ^{(r+1)}+\frac{1}{2}\Lambda_{m}, \end{aligned}$$

for $x\in \mathbb{Z}$.

Now, we can state our second result.

### Theorem 4.2

*For each integer* $l\geq2$, *let*

$$ \Lambda_{l}=\sum_{k=1} ^{l-1} \frac{1}{k(l-k)}b_{k}\mathbb {B}_{l-k}^{(r+1)}+2\sum _{k=1} ^{l-1}\frac{1}{k(l-k)}b_{k}{ \mathbb {B}}_{l-k-1} ^{(r)}, $$

*with* $\Lambda_{1}=0$. *Assume that* $\Lambda_{m}\neq0$ *for an integer* $m\geq 2$. *Then we have the following*.

- $$\begin{aligned}& =\frac{1}{m} \biggl(\Lambda_{m+1}-\frac{1}{m(m+1)}{ \mathbb {B}}_{m}^{(r)}-\frac {1}{m(m+1)}b_{m+1} \biggr) \\& \quad{}+\sum_{\substack{n=-\infty\\n\neq0}} ^{\infty} \Biggl\{ - \frac {1}{m}\sum_{s=1} ^{m} \frac{(m)_{s}}{(2\pi in)^{s}} \biggl(\Lambda_{m-s+1}+\frac {H_{m-1}-H_{m-s}}{m-s+1} \bigl({ \mathbb{B}}_{m-s} ^{(r)}+b_{m-s+1} \bigr) \biggr) \Biggr\} e^{2\pi inx} \\& = \textstyle\begin{cases} \sum_{k=1} ^{m-1}\frac{1}{k(m-k)}b_{k}( \langle x \rangle ){\mathbb{B}}_{m-k} ^{(r+1)}( \langle x \rangle),& \textit{for }x\notin {\mathbb{Z}},\\ \sum_{k=1} ^{m-1}\frac{1}{k(m-k)}b_{k}{\mathbb{B}}_{m-k} ^{(r+1)}+\frac{1}{2}\Lambda_{m},& \textit{for } x\in{\mathbb{Z}}. \end{cases}\displaystyle \end{aligned}$$
- $$\begin{aligned}& \frac{1}{m}\sum_{s=0} ^{m}\binom{m}{s} \biggl(\Lambda_{m-s+1}+\frac {H_{m-1}-H_{m-s}}{m-s+1} \bigl({\mathbb{B}}_{m-s} ^{(r)}+b_{m-s+1} \bigr) \biggr)B_{s}\bigl( \langle x \rangle\bigr) \\& \quad=\sum_{k=1} ^{m-1}\frac{1}{k(m-k)}b_{k} \bigl( \langle x \rangle \bigr){\mathbb{B}}_{m-k} ^{(r+1)}\bigl( \langle x \rangle\bigr), \quad\textit{for } x\notin{\mathbb{Z}}; \\& \frac{1}{m}\sum_{\substack{s=0\\s\neq1}} ^{m} \binom{m}{s} \biggl(\Lambda _{m-s+1}+\frac{H_{m-1}-H_{m-s}}{m-s+1} \bigl({ \mathbb{B}}_{m-s} ^{(r)}+b_{m-s+1} \bigr) \biggr)B_{s}\bigl( \langle x \rangle\bigr) \\& \quad=\sum_{k=1} ^{m-1}\frac{1}{k(m-k)}b_{k}{ \mathbb{B}}_{m-k} ^{(r+1)}+\frac{1}{2}\Lambda_{m}, \quad \textit{for } x\in{\mathbb{Z}}. \end{aligned}$$

## Results and discussion

In this paper, we study three types of sums of products of ordered Bell and poly-Bernoulli functions and derive their Fourier series expansion. In addition, we express those functions in terms of Bernoulli functions. The Fourier series expansion of the ordered Bell and poly-Bernoulli functions are useful in computing the special values of the poly-zeta and multiple zeta function. For details, one is referred to [3, 7–18]. It is expected that the Fourier series of the ordered Bell functions will find some applications in connection with a certain generalization of the Euler zeta function and the higher-order generalized Frobenius-Euler numbers and polynomials.

## Conclusion

In this paper, we considered the Fourier series expansion of the ordered Bell and poly-Bernoulli functions which are obtained by extending by periodicity of period 1 the ordered Bell and poly-Bernoulli polynomials on $[0, 1)$. The Fourier series are explicitly determined.
